# Development of algorithms to identify individuals with Neurofibromatosis type 1 within administrative data and electronic medical records in Ontario, Canada

**DOI:** 10.1186/s13023-022-02493-5

**Published:** 2022-08-26

**Authors:** Carolina Barnett, Elisa Candido, Branson Chen, Priscila Pequeno, Patricia C. Parkin, Karen Tu

**Affiliations:** 1 Elisabeth Raab Neurofibromatosis Clinic, Toronto General Hospital, University of Toronto, 200 Elizabeth St. 5EC Room 334, Toronto, ON M5G 2C4 Canada; 2 Institute of Health Policy, Management and Evaluation, University of Toronto, Toronto, Canada; 3 Division of Neurology, Department of Medicine, University of Toronto, Toronto, Canada; 4 ICES, Toronto, Canada; 5 Department of Pediatrics, Hospital for Sick Children, University of Toronto, Toronto, Canada; 6 North York General Hospital, Toronto, Canada; 7 Department of Family and Community Medicine, University of Toronto, Toronto, Canada; 8 Toronto Western Hospital Family Health Team, University Health Network, Toronto, Canada

**Keywords:** NF1, EMR, Administrative database, Algorithm

## Abstract

**Background:**

There is limited population-based data on Neurofibromatosis type 1 (NF1) in North America. We aimed to develop and validate algorithms using administrative health data and electronic medical records (EMRs) to identify individuals with NF1 in Ontario, Canada.

**Methods:**

We conducted an electronic free-text search of 15 commonly-used terms related to NF1 in the Electronic Medical Records Primary Care Database. Records were reviewed by two trained abstractors who classified them as confirmed, possible, and not NF1. An investigator with clinical expertise performed final NF1 classification. Patients were classified as confirmed if there was a documented diagnosis, meeting NIH criteria. Patients were classified as possible if (1) NF1 was recorded in the cumulative patient profile, but no clinical information to support the diagnosis; (2) only one criterion for diagnosis (e.g. child of confirmed case) but no further data to confirm or rule out. We tested different combinations of outpatient and inpatient billing codes, and applied a free-text search algorithm to identify NF1 cases in administrative data and EMRs, respectively.

**Results:**

Of 273,440 eligible patients, 2,058 had one or more NF1 terms in their medical records. The terms “NF”, “café-au-lait”, or “sheath tumour” were constrained to appear in combination with another NF1 term. This resulted in 837 patients: 37 with possible and 71 with confirmed NF1. The population prevalence ranged from 1 in 3851 (confirmed NF1) to 1 in 2532 (possible and confirmed NF1). Billing code algorithms had poor performance, with overall low PPV (highest being 71%). The accuracy of the free-text EMR algorithm in identifying patients with NF1 was: sensitivity 85% (95% CI 74–92%), specificity 100% (95% CI 100–100%), positive predictive value 80% (95% CI 69–88%), negative predictive value 100% (95% CI 100–100%), and false positive rate 20% (95% CI 11–33%). Of false positives, 53% were possible NF1.

**Conclusions:**

A free-text search algorithm within the EMR had high sensitivity, specificity and predictive values. Algorithms using billing codes had poor performance, likely due to the lack of NF-specific codes for outpatient visits. While NF1 ICD-9 and 10 codes are used for hospital admissions, only ~ 30% of confirmed NF1 cases had a hospitalization associated with an NF1 code.

## Introduction

Neurofibromatosis type 1 (NF1) is one of the most common autosomal dominant disorders, with minimum birth incidence estimated at 1 in 2500 births, and a prevalence of approximately 1 in 3000 to 4000 people [1]. NF1 is a multi-systemic disorder, affecting the skin in virtually all individuals. The most common manifestations of NF1 are cutaneous, including cafe-au-lait macules, axillary and inguinal freckling, and neurofibromas [2]. Cutaneous neurofibromas are peripheral nerve sheath tumors, which despite being benign can cause pain, discomfort, disfiguration, and are a major cause of anxiety and reduced quality of life [2, 3]. Up to 50% of individuals with NF1 have plexiform neurofibromas [4]; these, beyond causing disfigurement and pain, are associated with potential for transformation to malignant peripheral nerve sheath tumors (MPNST), which have a high mortality rate [5]. In addition to the risk of MPNST, individuals with NF1 have a higher risk of other malignancies, including optic glioma and brain tumors, breast cancer, gastrointestinal stromal tumors, and pheochromocytoma, among others [2].

Population-based studies provide an opportunity to study how individuals use the health care system, and can provide insights into outcomes and association with other diseases. This is especially useful in rare diseases, such as NF1, where some outcomes may not be observable in small cohort studies. Some population-based studies have relied on diagnostic codes associated with billings, typically ICD-9 or ICD-10, to identify NF1 individuals [6, 7]. However, most studies have not validated case ascertainment using medical records, so there is a high risk of misclassification. For example, there may be clerical errors in coding, or sometimes a code may be used when there is a suspicion of a disease that is later ruled out. Therefore, it is important to develop validated algorithms to correctly identify individuals with the disease. Another approach to conduct population-based research in NF1 is creation of a registry, such as the Finnish registry, that was created through an extensive search through medical records in the country, to identify individuals with NF1. This registry is linked with several healthcare databases, and has provided rich insights into population-based outcomes of individuals with NF1 [1, 8].

The Canadian single-payer system is managed at the provincial level, meaning that each province has its own set of coverage rules and a specific health-insurance provider. In Ontario, Canada’s most populous province, the Ontario Health Insurance Plan (OHIP) covers ≥ 95% of the more than 14 million residents of the province. In this large population, approximately 3 times the population of Finland, it is not feasible to identify all individuals with NF1 through extensive medical record search. However, it might be feasible to create a population-based cohort using health administrative data [9, 10].

We aimed to develop and validate algorithms to identify individuals with NF1 living in Ontario, Canada, using administrative data, as well as in primary care electronic medical records (EMRs). Once developed, these algorithms can help to study population-based outcomes and healthcare utilization in this population. As secondary outcome, we aimed to study prevalence of NF1 in the province of Ontario, Canada.

## Methods

### Data source and population

We used the Electronic Medical Record Primary Care (EMRPC) database (formerly known as EMRALD) to identify individuals with NF1 for algorithm development. EMRPC is a database that consists of *all* clinically relevant information from family physician electronic medical records (EMRs). EMRPC contains data from almost 400 family physicians and ~ 500,000 patients distributed throughout Ontario. Affiliated physicians participate on a voluntary basis, using TELUS Practice Solutions^®^ EMR software. EMRPC allows free text search in all available data, including a cumulative patient profile (CPP) which has a problem list, past medical history, family history, allergies, immunizations, and risk factors. EMRPC also includes family physician progress notes, specialist consultation reports, diagnostic tests, discharge summaries, laboratory tests and prescriptions. In general, the population enrolled in EMRPC is considered representative of the entire Ontario population [11].

#### Case determination

We considered EMRPC patients eligible if they were rostered (i.e. patients registered with a participating family physician or family practice to provide their primary care), with a valid health insurance number, had a clinical encounter within the past 2 years, and were on the EMR for ≥ 1 year. We then conducted an automated free-text search in all the EMR data available, including the CPP, but also progress notes and specialist letters, for all eligible patients, to identify potential NF1 individuals. We used a liberal search for the following terms: “NF”, “NF1”, “neurofibromatosis”, “neurofibroma”, “café-au-lait”, “Lisch”, “plexiform”, “axillary freckling”, “inguinal freckling”, “peripheral nerve sheath tumor”, “malignant peripheral nerve sheath tumor”, “MPNST”, and “optic glioma”. We examined the full patient chart from first entry, to the date of last data extraction, no later than March 31, 2016.

Two trained chart abstractors reviewed flagged charts to classify them as: “Confirmed NF1”—if there was a note from a specialty clinic or genetic test result confirming the diagnosis—“possible NF1” if the abstractors were uncertain of the diagnosis, “Not NF1”— if NF1 was clearly ruled out by a specialist, and “blank” if the chart made absolutely no mention to NF1. We tested intra-rater reliability of the chart abstractors, whereby 5% of the reviewed charts were selected at random, and were presented again to the original abstractors, blinded to their initial classification. To test inter-rater reliability, another random sample of 5% of the charts that were reviewed by one abstractor was then presented to the other abstractor and vice versa.

To validate the classification, one investigator with expert clinical knowledge of NF1 (CB) reviewed all charts initially classified as “confirmed” and “possible” and reclassified as confirmed, possible and not NF1. Cases were classified as confirmed if there was a diagnosis of NF1 based on 1988 NIH criteria [12], or if there was positive genetic testing with consistent clinical manifestations. Cases were classified as possible if there was a suspicion of NF1, but insufficient data on the charts to rule it in or out. For example, young children with ≥ 6 cafe-au-lait spots who are too young to have other NF1 manifestations; or children of an individual with confirmed NF1, but without specific assessments in the EMR to assess clinical NF1 manifestations. Cases were also classified as possible if there was a diagnosis of NF1 in the CPP but without any clinical records describing NF1 specific manifestations or concerns, to confirm the diagnosis. All other records were classified as not NF1.

The patients classified as confirmed NF1 were considered the true positives for the algorithms; all other patients in the EMRPC database were considered non-NF1.

#### Algorithm development

Physicians submit claims to OHIP for each clinical encounter either through fee-for-service or shadow billing; these are submitted with a corresponding diagnostic code. Each person insured by OHIP has a unique 10-digit health insurance number which is encrypted and used for linkage to different ICES databases. This includes a variety of administrative and other health-related data, including OHIP data for physician services (from 1991), and hospital services such as hospitalizations, emergency department visits and day surgery. These databases are linked together using unique encoded identifiers and analyzed at ICES.

We surveyed health providers who routinely treat patients with NF1, to develop a list of commonly used OHIP billing codes and procedures. These are the codes used for billing outpatient services. For hospital services we used the Canadian Institute for Health Information (CIHI) database that contains discharge diagnosis on the discharge abstract database (DAD); we also used diagnostic codes within the same day surgery (SDS) data from the National Ambulatory Care Reporting System (NACRS) database. These databases use ICD-9 codes for services before 2002 and ICD-10 thereafter. After identifying documented NF1 cases in EMRPC through chart abstraction, we assessed all outpatient and inpatient billing codes in the patients with confirmed NF1. For the algorithm testing, we chose billing codes that had previously been identified by specialists treating NF1 individuals, and that also were present in the list of highly used billing codes for the confirmed NF1 cases.

We tested different combinations of the selected outpatient (OHIP) and hospital-related diagnostic codes (CIHI, NACRS and SDS databases), with different timeframes (e.g. ever billed or billed ≥ 1, 2 or 3 times in 1, 2 and 3 years). We also included algorithms that specified the specialties associated with the outpatient codes, focusing on specialties that frequently care for individuals with NF1 in the province (e.g. neurology, neurosurgery, paediatrics, plastic surgery, dermatology).

We also tested the performance of a free-text search on the EMR to accurately identify individuals with NF1, using NF1-related terms in the problem list and past medical history sections of the CPP. For all algorithms we calculated sensitivity, specificity, positive and negative predictive values (PPV and NPV) with 95% confidence intervals (CI).

#### Sample size

We used nomograms for sample size estimation for diagnostic accuracy. With a predicted prevalence of 1 in 4000 (0.00025), for 90% sensitivity and specificity, and accuracy (half-width of the confidence interval) of 0.05, a minimum of 10,000 individuals (cases and non-cases) were needed [13].

#### Ethics

ICES is a prescribed entity under Ontario’s Personal Health Information Protection Act (PHIPA). Section 45 of PHIPA authorizes ICES to collect personal health information, without consent, for the purpose of analysis or compiling statistical information with respect to the management of, evaluation or monitoring of, the allocation of resources to or planning for all or part of the health system. Projects that use data collected by ICES under section 45 of PHIPA, and use no other data, are exempt from REB review. At ICES, all datasets are encoded in a process that removes direct personal identifiers to ensure patient confidentiality. The use of the data in this project is authorized under section 45 and approved by ICES’ Privacy and Legal Office.

## Results

### Chart abstraction

**Fig. 1:**
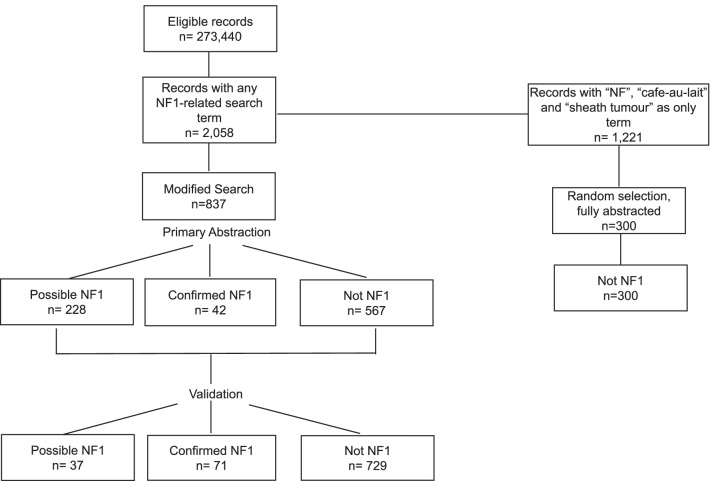
Chart abstraction and validation. Figure depicts the process of chart abstraction and validation, after initial search in 273,440 eligible records from the EMRCP database

At the time of the search (January 2019), EMRPC database had 273,440 patients that met eligibility criteria. The mean age was 43.9 ± 23 years and 55.8% were female. Mean time on the EMR was 6.45 ± 3.5 years. On the initial search, 2058 records had at least one search term. While pilot-testing the abstraction platform, we found that the terms: “NF”, “Cafe-au-lait” and “sheath tumour” when found alone, were not related to NF1. For example, “NF” usually referred to the Canadian province of Newfoundland or the verification code of the health card. Therefore, we modified the search strategy so that these terms had to be found with at least another search term to flag the record. This modified search resulted in 837 patients whose charts were fully abstracted. The trained abstractors marked 42 patients as confirmed NF1, 228 as possible (confirmed and possible n = 270) and 567 as “no NF1” or “blank” (no mention of NF1). Figure 1 depicts the chart abstraction and validation process. The inter and intra-rater agreement between chart abstractors was high, between 96 and 100%.

Validation of the 270 records flagged as confirmed and possible yielded 71 confirmed NF1 cases and 37 possible NF1 cases (confirmed and possible n = 108); the remaining 162 were re-classified as non NF1 for a total of 729 non-NF1 individuals. To assess the validity of the modified search strategy, we also abstracted 300 random charts, 100 for each of the terms we excluded when present alone (‘Cafe-au-lait”, “NF”, “sheath tumour”). After validation, none of these records corresponded to NF1 cases. The minimum prevalence of NF1 in this population was 1 in 3851 (95% CI 1 in 3107 to 1 in 4921), considering the confirmed cases only, and 1 in 2532 (95% CI 1 in 2183 to 1 in 3038) considering both confirmed and possible cases.

#### Diagnostic algorithms

From the 71 confirmed NF1 cases, we identified the following OHIP billing codes (outpatient services) that were also in the billing list from specialists: 192—“malignant tumours of the cranial nerves or spinal cord”, 216—“skin lesion: e.g. dermatofibroma”, 225—“ benign tumours of the brain, spinal cord or peripheral nerves”, 709—“other disorders of the skin and subcutaneous tissue” and 758—“chromosomal abnormalities”. ICD-9 codes were 237.7: “NF unspecified” and 237.71: “NF1”. The ICD-10 code for NF is Q85.0. Only 22 of the 71 (31%) patients with confirmed NF1 had a hospital-related encounter (hospitalization, emergency department visit or same day surgery) with an NF1 diagnostic code (ICD-9 or ICD-10).

We tested several administrative algorithms in a reference cohort of 273,402 eligible individuals within EMRPC, of which 71 were confirmed NF1. The demographic characteristics of this cohort are in Table 1. Overall, these algorithms had poor performance; the highest PPV was 71%, but sensitivity was only 21%, with 79% false negatives. Table 2 depicts the most relevant algorithms tested and their performance. We then assessed the performance of a free-text search strategy within the EMR to identify the true cases within the initial eligible sample of 273,440 records. Patients were considered positive if those fields had mention of “NF1”, “Recklinghausen”, or “neurofibromatosis” (and various misspellings), unless the disease term was in close proximity to an exclusion word/phrase (e.g. “no sign of”, “screen for”, “not”, “unconfirmed”, “NF2”). Specific search terms of this algorithm are summarized in Table 3. This algorithm resulted in a sensitivity 85% (95% CI 74–92%), specificity 100% (95% CI 100–100%), positive predictive value (PPV) 80% (95% CI 69–88%), negative predictive value 100% (95% CI 100–100%), and a false positive rate of 20% (95% CI 11–33%). Of the 15 false positives, 8 (53%) were possible NF1 cases.

**Table 1:** Demographic characteristics of eligible EMRPC individuals in the cohort used for algorithm development

	Definitive NF1 N = 71	Possible NF1 N = 37	Not NF1 N = 273,331
Age (mean ± SD)	39.3 ± 23.0	41.1 ± 24.8	43.9 ± 23.0
Sex (female)	36 (50.7%)	19 (51.4%)	152,514 (55.8%)
Follow up time, years (mean ± SD)	7.09 ± 3.2	8.97 ± 5.3	6.45 ± 3.5
Rural	10 (14.1%)	8 (21.6%)	49,464 (18.1%)
Urban	61 (85.9%)	29 (78.4%)	222,819 (81.5%)
Income quintile 1	14 (19.7%)	13 (35.1%)	44,464 (16.3%)
Income quintile 2–3	24 (33.8%)	10 (27.0%)	102,704 (37.6%)
Income quintile 4	14 (19.7%)	6 (16.2%)	50,024 (21.2%)
Income quintile 5	19 (26.8%)	8 (21.6%)	66,778 (24.4%)

Income quintile using nearest census based neighbourhood

*EMRPC* electronic medical record database

**Table 2:** Performance of selected algorithms to identify individuals with NF1

Algorithm	True positive	True negative	False negative	False positive	Sensitivity (95% CI)	Specificity (95% CI)	PPV (95% CI)	NPV (95% CI)
EMR search	60	273,354	11	15	85 (74–92)	100 (100–100)	80 (69–88)	100 (100–100)
Billing algorithms								
CIHI	15	273,325	56	6	21 (12–31)	100 (100–100)	71 (52–91)	100 (100–100)
CIHI or NARCS or SDS	22	273,321	49	10	31 (20–42)	100 (100–100)	69 (53–85)	100 (100–100)
1 OHIP ever	65	125,703	6	147,628	92 (85–98)	46 (46–46)	0 (0–0.1)	100 (100–100)
CIHI or 2 OHIP* ever	58	218,349	13	54,982	82 (73–91)	80 (80–80)	0.1 (0.1–0.1)	100 (100–100)
CIHI or 4 OHIP in 3 y	45	246,079	26	27,252	90 (90–90)	63 (52–75)	0.2 (0.1–0.2)	100 (100–100)
OHIP 225 & OHIP 758* ever	6	273,319	65	12	9 (2–15)	100 (100–100)	33 (12–55)	100 (100–100)
CIHI or 2 OHIP* ever	55	221,560	16	51,771	70 (58–80)	81 (91–81)	0.1 (0.1–0.2)	100 (100–100)

OHIP codes included: 192, 216, 225, 709, 758

*CIHI* Canadian Institute for Health Information, *EMR* electronic medical record, *NARCS* national ambulatory care reporting system

*Code billed by selected specialties (neurology, neurosurgery, pediatrics, genetics, plastic surgery and general surgery)

**Table 3:** Cumulative patient profile search terms for identification individuals with NF1 from EMR database

Search inclusion terms	Selected exclusion terms
‘NF1', 'NF1', 1	‘negative for' or ‘no evidence’ or ‘no history of'
'NF1[^a-z0-9]%', 1	‘at risk of’ ‘?early’
'NF1', '%[^a-z0-9]NF1', 1	'family history of' or 'FMHx' or ‘fhx’
'NF1', '%[^a-z0-9]NF1[^a-z0-9]%', 1	'without' ‘with out'
'recklinghausen', '%recklinghausen%', 1	‘maternal’ or ‘paternal’ or ‘brother’ or ‘sister’
'neurofibromatosis', '%neurofibr_m_tos_s%', 1	'unconfirmed' 'suspect'
'neurofibromatosis', '%Neuro Fibromatosis%', 1	'rule out' or 'r/ o' or 'r/o'
'neurofibromatosis', '%Neurofibiromatosis%', 1	‘possible’ or ‘possibly’ or ‘probable’
'neurofibromatosis', '%neurofibomatosis%', 1	‘NF2’

#### Assessing billing patterns on EMR databases

From the confirmed NF1 records identified in EMRPC, we identified the most commonly used outpatient diagnostic codes, as seen in Table 4. Approximately 35% of outpatient encounters did not have a specific diagnostic code. The most common diagnostic code was code 304: drug dependence/drug addiction, which was billed 1216 times (4.3% of clinical encounters). When combining all codes related to skin diagnoses, these were billed 822 times (2.9% of clinical encounters), The remaining diagnostic codes were each billed in 2% or less encounters.

**Table 4:** Selected commonly used OHIP outpatient diagnostic billing codes in 71 patients with confirmed NF1 and 28,409 clinical encounters

Codes	Description	Frequency	Percent of codes
999	No diagnosis	9838	34.6
304	Drug dependence/drug addiction	1216	4.3
709 or	Other disorders of skin and subcutaneous tissue	822	2.9
216 or	Dermatofibroma, pigmented nevus		
173	Other skin malignancies		
787	Anorexia/nausea and vomiting	605	2.1
781	Leg cramps, pain (muscle, joints)	372	1.3
300	Anxiety, depression	347	1.2
192	Malignant tumours of the nervous system outside of brain	278	1.0
349	Other diseases of the central nervous system	266	0.9
401	Essential hypertension	241	0.9
758	Autosomal anomalies, chromosomal anomalies	141	0.5
225	Benign tumours of the nervous system	102	0.4
191	Malignant brain tumours	98	0.3
759	Other congenital anomalies	95	0.3
313	Behaviour disorders of childhood and adolescence	60	0.2
315	Developmental delay	45	0.2

Codes 709, 216 and 173 all represent common skin-related issues in NF1, and therefore were combined for these analyses

## Discussion

In this study, we identified records of individuals with confirmed NF1 within a large, population-based database of EMRs. We found that a free-text search in the cumulative patient profile of primary care EMRs is a simple, yet effective way of identifying individuals with NF1. Because NF1 is a multisystemic disease where patients are often seen by different specialties, being able to identify people with NF1 from primary care records may provide more complete information than records from single specialties. Additionally, primary care records may be more reflective of the general population compared to a single specialty centre or clinic. This simple search algorithm can be used in the future to study other outcomes in other databases, so there is potential for generalizability; however, this algorithm should be validated in other EMRs before use, as the way physicians document the diagnosis of NF1 may differ in different healthcare settings. Because the uptake of EMRs is increasing, we believe that in the future this algorithm can be adapted to different settings to identify larger numbers of individuals living with NF1.

Our administrative data algorithms using physician billing codes performed poorly and were not able to accurately identify individuals with NF1. There are several reasons for this. First, there is no specific NF1 diagnostic code in OHIP for outpatient visits. Therefore, clinicians use codes that apply to their specialty practices, such as codes for skin lesions in the case of dermatology/plastic surgery, brain tumours in the case of neurosurgery, or codes for general genetic diseases for other specialties. We had hypothesized that a combination of these codes as well as the specific medical specialties could help identify individuals with NF1. For example, we thought that NF1 patients would have codes for specific skin lesions in combination with codes for genetic diseases, plus visits by certain specialties (e.g. neurology/neurosurgery). However, algorithms that followed this logic resulted in high numbers of false positives. The best performing algorithms were those related to hospital services that use ICD codes. However, only 22 out of the 71 confirmed cases had a hospital-related billing (including same day surgery) associated with an NF1 code. It is not clear if this reflects few NF1-related hospital services in this cohort, or lack of awareness of NF1 by healthcare providers, whereby NF1 was not identified as a main health problem for a given admission.

Our estimate of minimum prevalence in this population is within published ranges; however, this is likely an underestimate of the true prevalence. For example, if the 37 probable cases were confirmed, the prevalence would increase from 1 in 3,851 to 1 in 2,532. Additionally, in our study we only identified individuals who had a diagnosis of NF1, but previous work has shown that with detailed screening programs on NF1 manifestations, the prevalence can be as high as 1 in 1,000, identifying many individuals without a previous diagnosis [14, 15]. This may be due to some individuals with very mild clinical features who may not receive a formal diagnosis of NF1.

A surprising finding from this study was that the diagnostic billing code for drug addiction/dependency was the most commonly used outpatient code in the 71 confirmed cases, accounting for 4.3% of clinical encounters. As a comparison, in previous studies from the general population of Ontario, drug addiction was not in the top 10 diagnostic codes [16]. Further work is needed to assess the prevalence of substance dependence in larger population-based studies of individuals with NF1, to determine if this finding is replicated in other settings. Because cutaneous manifestations of NF1 are highly prevalent in NF1 (> 90% of individuals), and given the absence of specific NF1 diagnostic codes, we combined the 3 most commonly used skin-related outpatient diagnostic codes used in confirmed NF1 cases. With this approach, these became the second most common outpatient diagnostic code accounting for 2.9% of all billings. Further work is needed to understand the healthcare utilization patterns of individuals living with NF1.

This study has some limitations. Our findings are biased towards individuals with NF1 who actively use the healthcare system, so we will have missed those with mild disease who do not seek care, as well as very young children without clinical manifestations, and also those who have limited access to healthcare due to social or geographical reasons. Our chart abstraction strategy was comprehensive, but it is possible that some individuals with clinically evident NF1 who do use the healthcare system, do not have their NF1 clinical manifestations and/or diagnosis recorded in the medical records. These individuals would not have been flagged in our search of the EMR. Despite these limitations, we found that our EMR search strategy performs well to identify individuals with a clinical diagnosis of NF1.

The generalizability of this strategy needs to be assessed and validated in other settings, for example in databases that use a different EMR vendor. The use of a free-text search makes generalizability more likely across vendors, because it can identify the search terms regardless of where they are stored in the EMR or how the EMR is organized. However, this strategy will still need to be validated in a different EMR. It is also possible that some clinicians use other terms or abbreviations (e.g. CALMs for cafe-au-lait macules) that may need to be incorporated in the search terms when validating this search strategy in other settings. Other limitations to generalizability include language, as our search strategy was done in English, therefore language and cultural-specific search terms will be needed in different regions. Finally, we do acknowledge that electronic medical records will be limited or non-existent in low income regions, where this approach may not be feasible. However, we believe that any effort to identify cohorts of patients with rare diseases, such as NF1, where clinical questions can be answered, will eventually be useful to other patients, including those without access to electronic medical records.

In summary, we found that a simple EMR search is highly accurate in identifying individuals with NF1 from EMRs, and can be used for future studies. Administrative data algorithms within Ontario had poor performance therefore province-wide identification of NF1 using administrative data is not possible. However, linkage of registry data or EMR data to administrative databases can still help study health utilization and outcomes of individuals with NF1 in the province, where our estimate of NF prevalence is in keeping with published literature.

## Data Availability

The dataset from this study is held securely in coded form at ICES. While legal data sharing agreements between ICES and data providers (e.g., healthcare organizations and government) prohibit ICES from making the dataset publicly available, access may be granted to those who meet pre-specified criteria for confidential access, available at www.ices.on.ca/DAS (email: das@ices.on.ca). The full dataset creation plan and underlying analytic code are available from the authors upon request, understanding that the computer programs may rely upon coding templates or macros that are unique to ICES and are therefore either inaccessible or may require modification.
